# Patient advice regarding participation in sport in children with disorders of cerebrospinal fluid (CSF) circulation: a national survey of British paediatric neurosurgeons

**DOI:** 10.1007/s00381-020-04536-3

**Published:** 2020-02-26

**Authors:** M. Zaben, S. Manivannan, C. Petralia, I. Bhatti, C. Patel, P. Leach

**Affiliations:** 1 Department of Paediatric Neurosurgery, University Hospital of Wales, Cardiff, UK; 2 Neuroscience and Mental Health Research Institute (NMHRI), School of Medicine, Cardiff University, Room 4FT 80E, 4th Floor, University Hospital Wales, Heath Park, Cardiff, CF14 4XN UK

**Keywords:** Paediatric neurosurgery, Sports, Disorders of CSF circulation, Ventriculoperitoneal shunt, Chiari malformation, Arachnoid cyst

## Abstract

**Background:**

Management of children with disorders of cerebrospinal fluid (CSF) circulation is a common aspect of paediatric neurosurgical practice. Sport and physical activity play an integral role in the lives of patients in this age group. However, there is little evidence to support the dissemination of appropriate advice to children regarding such activities. The aim of this study was to evaluate the perspectives of clinicians across the UK regarding the participation of children with disorders of CSF circulation in sports.

**Methods:**

Questionnaires were distributed to Consultant Paediatric Neurosurgeons practising across the UK via the Society of British Neurological Surgeons (SBNS). Five different patient scenarios were supplied, and participants were asked to choose whether they would advise participation in the following sports: Taekwondo, rugby, skiing, and football.

**Results:**

An overall response rate of 66.7% (36 out of 54 paediatric neurosurgeons) was achieved. The following percentages of clinicians advocated football, rugby, Taekwondo, and skiing across all scenarios: 96%, 75%, 77%, and 97%, respectively. The majority of responders (91.2%) relied on personal experience when providing advice, whilst 50% used available literature and 19.4% used available guidelines.

**Conclusions:**

There is a paucity of evidence in the literature to support the dissemination of appropriate advice to children with disorders of CSF circulation regarding participation in sports. Our findings demonstrate that the majority of clinicians rely on personal experience to make such decisions, emphasizing the necessity of larger scale studies to inform evidence-based guidelines.

## Introduction

Managing children with disorders of cerebrospinal fluid (CSF) circulation is common in paediatric neurosurgical practice. The importance of physical activity and sport for maintaining both physical and mental health are widely acknowledged [1, 2]. Its relevance to paediatric cohorts is highlighted by increasing rates of childhood obesity and its potential to reduce the incidence of depression and anxiety in children [1, 2]. Given the issues that children with disorders of CSF circulation must live with, it is vital that normality of life is achieved in every domain possible. Therefore, one of the most common questions arising from these children and their parents relates to whether it is safe to participate in certain sporting activities. In the absence of level one evidence and/or well-defined guidelines, giving appropriate advice remains a hit-and-miss exercise. Few studies have addressed this issue, mainly focusing on singular sporting activities or specific pathologies [3–5]. Given the paucity of evidence to support the dissemination of appropriate advice to patients, we aimed to identify perspectives of paediatric neurosurgeons across the UK. Therefore, in this study, we performed a nationwide survey of paediatric neurosurgeons to elucidate current practice regarding recommendations being given to children with disorders of CSF circulation who wish to participate in sport activities.

## Methods

**Fig. 1:**
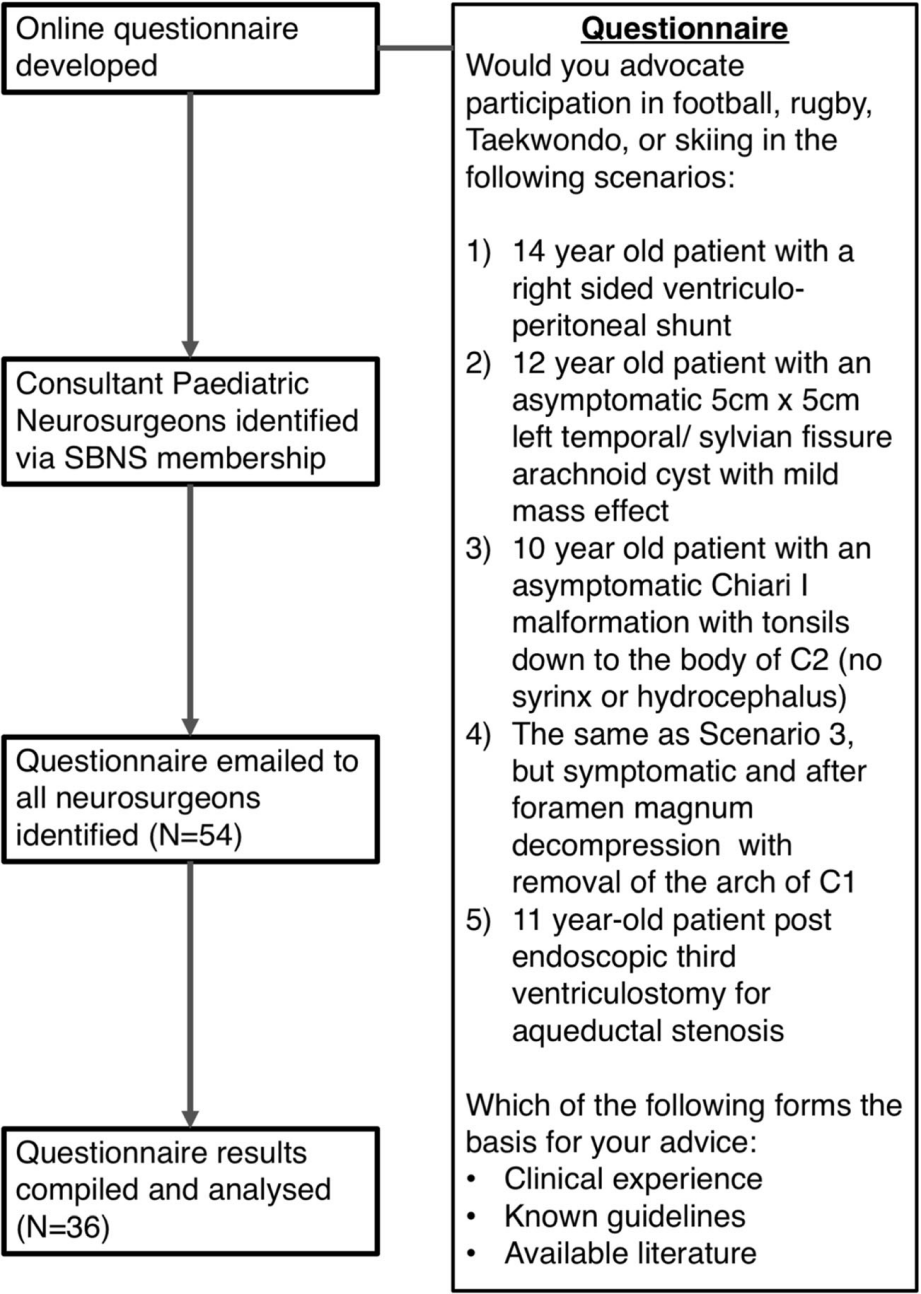
Summarizes strategy for national survey of paediatric neurosurgeons in UK

Practising paediatric neurosurgeons across the UK were identified via the Society of British Neurological Surgeons (SBNS) member’s database. A total of 54 paediatric neurosurgeons were identified, and online questionnaires were distributed to them between June 2018 and June 2019. The survey consisted of six questions (see Fig. 1). Five binary response questions involved different paediatric clinical scenarios and whether clinicians would advocate participation in four different contact sports (Taekwondo, rugby, skiing, and football). The final multiple-choice question explored the rationale behind the decisions made in the previous questions. A multi-database (PubMed, Ovid) literature review was performed to identify articles discussing sport participation in children with disorders of CSF circulation on 17th July 2019. Our search strategy involved varying combinations of the following search terms: “Chiari malformation”, “arachnoid cyst”, “ventriculoperitoneal shunts”, “aqueductal stenosis”, “endoscopic third ventriculostomy”, “athletics,” “physical activities,” “limitations”, and “sport”.

## Results

A total of 54 consultant paediatric neurosurgeons practising in the UK were identified via the SBNS and asked to complete an online survey. An overall response rate of 66.7% (36 out of 54 neurosurgeons) was achieved (Fig. 1).

### Case scenario 1

**Fig. 2:**
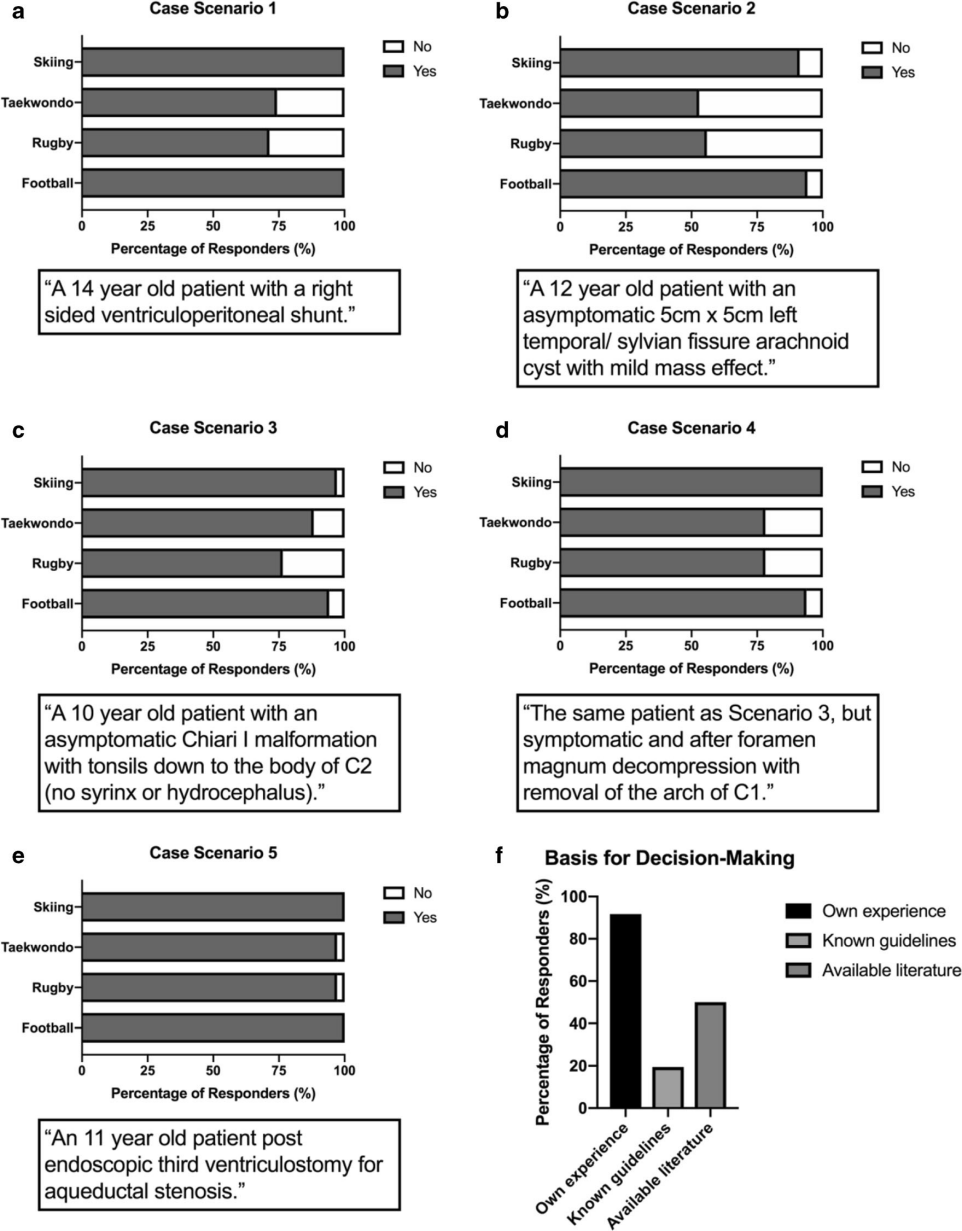
Summarizes the results of the online questionnaire. Opinions of respondents regarding participation in four different sports based on clinical scenarios (a–e), and the rationale behind their decision-making (f)

This scenario regarding a 14-year-old patient with a ventriculoperitoneal (VP) shunt was answered by the majority of responders (97.2%; 35/36) (Fig. 2a). All responders agreed to allowing the patient to participate in football and skiing. However, only 71.4% (25/35) and 74.3% (26/35) advocated participation in rugby and Taekwondo, respectively.

### Case scenario 2

The majority of responders (94.4%; 34/36) answered this question regarding a 12-year-old patient with an asymptomatic arachnoid cyst (Fig. 2b). Football was recommended by all responders, whilst 91.2% (31/34) advised participation in skiing. Only 55.9% (19/34) and 53.0% (18/34) however felt that participation in rugby and Taekwondo was advisable, respectively.

### Case scenario 3

The question regarding this scenario regarding a 10-year-old patient with an asymptomatic Chiari I malformation and tonsils down to the body of C2 was completed by 94.4% (34/36) of responders (Fig. 2c). In contrast, 97.1% (33/34) and 94.1% (32/34) advocated participation in skiing and football, respectively. However, participation in rugby was still supported by 76.5% (26/34) and Taekwondo by 88.2% (30/34).

### Case scenario 4

This scenario was identical to the previous scenario, except the patient had undergone foramen magnum decompression due to symptoms (Fig. 2d). A lower response rate was observed, with 88.9% (32/36) responding. All responders supported participation in skiing and 93.4% (30/32) for football. Both rugby and Taekwondo were advocated by 78.1% (25/32) of responders.

### Case scenario 5

The majority of responders (97.2%; 35/36) answered this scenario regarding an 11-year-old patient that underwent an endoscopic third ventriculostomy (ETV) for aqueductal stenosis (Fig. 2e). All responders supported participation in both football and skiing, whilst 97.1% (34/35) advocated both rugby and Taekwondo.

### Decision-making rationale

The final question explored the rationale behind decisions made by responders, with the following options: personal experience, guidelines, and published literature (Fig. 2f). Responses were received by all participants, and the majority (91.2%; 33/36) reported basing decisions on their own experience. Half of responders (50%; 18/36) also reported using available literature, and a minority (19.4%; 7/36) used known guidelines.

## Discussion

Our study demonstrates that paediatric neurosurgeons predominantly rely on personal experience to issue advice regarding sport participation in children with disorders of CSF circulation, with little evidence for support. Current studies examining sports-associated complications are either pathology or sport-specific (Table 1).

**Table 1:** Summary of studies reporting sports participation in patients with disorders of CSF circulation

Study	Design	Cohort	Findings
Blount et al. 2004	Online national survey	Clinician experience with sport-associated complications in children with VP shunts	77% of clinicians have not experienced sports-associated complications
Meehan et al. 2015	Single-centre retrospective study	Sport participation in patients with Chiari malformations	No serious neurological injuries (death, coma, or paralysis)
Shastin et al. 2016	Literature review	Scuba-diving in patients with VP shunts	No associated complications
Strahle et al. 2016	Single-centre prospective study	Sport participation in children with arachnoid cysts	Two patients suffered subdural hygromas (no intervention required)
Strahle et al. 2016	Multi-centre prospective study	Sport participation inpatients with Chiari type 1 malformation	No serious injuries
Spencer et al. 2017	Literature review	Sport/trauma associated injuries in patients with Chiari type I malformation	Anecdotal evidence of sudden death following head or neck injury but not in larger cohort studies

Only one study examined sport-related complications in children with ventriculoperitoneal shunts by performing a national survey of paediatric neurosurgeons in the USA [5]. Interestingly, 77% of clinicians had never experienced sport-related shunt complications, and 90% did not restrict patients from participating in non-contact sports. On the other hand, one third of clinicians did not advocate any form of contact sport, whilst another third only restricted some contact sports. However, the applicability of this study is limited by the fact that a response rate of only 55% was achieved and does not reflect any potential changes in clinician attitudes over the ensuing 15 years. Nonetheless, these findings largely reflect our results, with all participating paediatric neurosurgeons advocating football and skiing, and more than 70% supporting participation in rugby and Taekwondo (Fig. 2a). More recent studies have evaluated specific neurosurgical pathologies and specific sporting activities. We have previously examined the safety of VP shunts when scuba diving and identified no reports of associated complications in the literature [3]. However, this was not specific to the paediatric population and did not evaluate other sports.

A recent single-centre prospective study in the USA examined sports participation in children with arachnoid cysts [4]. Patients were followed up over a 46-month period and of 112 patients that participated in sports, only two patients suffered symptomatic subdural hygromas. Interestingly, our survey demonstrates that clinicians were most conservative with advocating participation in sport in the case scenario involving an asymptomatic arachnoid cyst. Indeed, the lowest rates of clinician support for all four sports were observed in this scenario (Fig. 2b).

Three papers addressing sports participation in patients with Chiari malformations were identified [6–8]. One literature review examined the consequences of trauma or contact sports on patients with asymptomatic Chiari type I malformations [6]. Other than anecdotal evidence of sudden death in patients following head or neck injury, severe neurological deterioration was not identified in any studies of larger cohorts. A single centre retrospective cohort study in the USA examined the incidence of serious injuries in patients with Chiari malformations that participated in sports over a 3-year period [7]. Across 147 patients, there were no reports of death, coma, or paralysis with respect to sports participation. Another multi-centre prospective study in the USA evaluated sport-related injuries in patients with Chiari type I malformations [8]. Whilst only 65.2% (328 out of 503 patients) participated in sports, there were no reports of serious neurological injuries in these patients. Similar to our results with clinician opinions regarding arachnoid cysts, responders were relatively conservative, with only 76.5% and 88.2% advocating rugby and Taekwondo, respectively (Fig. 2c).

Given that arachnoid cysts and Chiari type I malformation are the only pathologies with clinical evidence indicating a positive safety profile, it is interesting that paediatric neurosurgeons remain relatively more restrictive in their attitudes towards sports participation when compared with the remaining clinical scenarios. Follow-up studies to examine whether clinicians have personal experience of sports-associated complications in patients with these pathologies may provide invaluable insight. There were no studies exploring the safety of sport participation in patients that underwent ETV or foramen magnum decompression (FMD) for Chiari malformation. Our results show that paediatric neurosurgeons have similar opinions regarding sport participation in patients with Chiari type I malformations, whether FMD was performed or not (Fig. 2 c and d). However, the lowest overall response rate (88.9%) was observed for the case scenario examining FMD. It is possible that this was a result of an absence of tangible evidence in the literature to support the opinions of clinicians. Importantly, we demonstrate that the majority of clinicians rely on personal experience to provide appropriate advice to patients (Fig. 2f). Larger scale studies examining the safety of sports participation in children with disorders of CSF circulation are required to develop evidence-based guidelines.

## Conclusion

There is a paucity of evidence in the literature to support the dissemination of appropriate advice to children with disorders of CSF circulation regarding participation in sports. Our findings demonstrate that the majority of paediatric neurosurgeons rely on personal experience to make such decisions, emphasizing the necessity of larger scale studies to inform evidence-based guidelines.
